# Extended ICI treatment after first‐line chemoimmunotherapy could predict the clinical benefit of ramucirumab plus docetaxel in advanced non‐small lung cancer: Post hoc analysis from NEJ051 (REACTIVE study)

**DOI:** 10.1111/1759-7714.15173

**Published:** 2023-11-27

**Authors:** Ou Yamaguchi, Keita Mori, Saori Takata, Kazuhiko Shibata, Kenichi Chikamori, Nozomu Kimura, Yoshiaki Nagai, Taku Nakagawa, Satoshi Igawa, Taishi Harada, Hiroshige Yoshioka, Hisashi Tanaka, Hitomi Nogawa, Hiroaki Satoh, Toshihiro Shiozawa, Kosuke Tsuji, Kunihiko Kobayashi, Kyoichi Kaira

**Affiliations:** ^1^ Department of Respiratory Medicine, Comprehensive Cancer Center Saitama Medical University International Medical Center Hidaka Japan; ^2^ Clinical Research Center Shizuoka Cancer Center Nagaizumi Japan; ^3^ Department of Respiratory Medicine Kyorin University Hospital Mitaka Japan; ^4^ Division of Medical Oncology, Department of Medicine Kouseiren Takaoka Hospital Takaoka Japan; ^5^ Department of Medical Oncology NHO Yamaguchi‐Ube Medical Center Ube Japan; ^6^ Department of Respiratory Medicine Tohoku University Graduate School of Medicine Sendai Japan; ^7^ Division of Respiratory Medicine, Clinical Department of Internal Medicine Jichi Medical University Saitama Medical Center Saitama Japan; ^8^ Department of Thoracic Surgery Omagari Kosei Medical Center Omagari Japan; ^9^ Department of Respiratory Medicine Kitasato University School of Medicine Sagamihara Japan; ^10^ Department of Respiratory Medicine Japan Community Health Care Organization Kyushu Hospital Fukuoka Japan; ^11^ Department of Thoracic Oncology Kansai Medical University Hirakata Japan; ^12^ Department of Respiratory Medicine Hirosaki University Graduate School of Medicine Hirosaki Japan; ^13^ Department of Respiratory Medicine Yamagata Prefectural Central Hospital Yamagata Japan; ^14^ Division of Respiratory Medicine, Mito Medical Center University of Tsukuba Mito Japan; ^15^ Department of Respiratory Medicine, Faculty of Medicine University of Tsukuba Tsukuba Japan; ^16^ Department of Respiratory Medicine, Faculty of Medicine Hokkaido University Sapporo Japan

**Keywords:** chemoimmunotherapy, ICI, lung cancer, maintenance therapy, predictive, ramucirumab plus docetaxel

## Abstract

**Background:**

The factors that predict the clinical response to ramucirumab plus docetaxel (RD) after first‐line chemoimmunotherapy are unresolved. We explored whether the therapeutic efficacy of prior chemoimmunotherapy could predict the outcome of RD as sequential therapy in patients with advanced non‐small cell lung cancer (NSCLC).

**Methods:**

Our study comprised 288 patients with advanced NSCLC who received RD as the second‐line treatment after first‐line chemoimmunotherapy at 62 Japanese institutions. Chemoimmunotherapy consisted of a platinum‐based regimen and immune checkpoint inhibitors (ICIs). The association between several variables and the therapeutic outcome of RD was determined via logistic regression analysis.

**Results:**

Of the 288 patients, 225 (78.1%) received maintenance therapy and 108 (37.5%) received both ICI treatment for >180 days and maintenance therapy. All of 108 patients having ICIs for >180 days received maintenance therapy. Univariate analysis identified performance status, histology (adenocarcinoma), maintenance therapy, and ICI treatment >180 days as significant predictors of better progression‐free survival (PFS) and overall survival (OS) after RD administration. Multivariate analysis confirmed that these factors independently predicted favorable PFS and OS. The therapeutic response and PD‐L1 expression were not closely associated with outcome after RD treatment. In particular, maintenance therapy >4 cycles was more predictive of the better prognosis for RD treatment.

**Conclusion:**

Extended ICI treatment after chemoimmunotherapy and maintenance therapy enhanced the efficacy of second‐line RD treatment in patients with advanced NSCLC.

## Graphical abstract

Extended checkpoint inhibitor (ICI) treatment after chemoimmunotherapy enhances the efficacy of second‐line ramucirumab plus docetaxel (RD) treatment. First‐line ICI treatment for >180 days was independent predictors of better outcomes after RD administration. Continuous administration of prior ICI for >160 days could definitely predict the PFS of >6 months for DR.

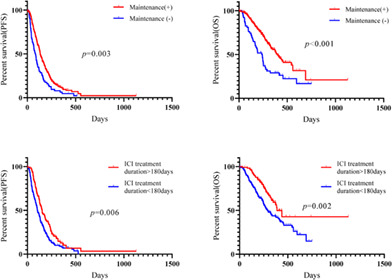

## INTRODUCTION

Advanced non‐small cell lung cancer (NSCLC) is an aggressive and difficult to eradicate disease. Recently, outcomes have improved significantly owing to the use of immune checkpoint inhibitors (ICIs), such as antiprogrammed death‐1 (PD‐1) and antiprogrammed death ligand‐1 (PD‐L1) antibodies.^1^ Chemoimmunotherapy consisting of a platinum‐based regimen plus anti‐PD‐1/PD‐L1 antibodies is more effective than is chemotherapy alone, even if the tumor cells do not express PD‐L1.^2^, ^3^, ^4^

After first‐line chemoimmunotherapy, cytotoxic agents (e.g., docetaxel) are usually administered to patients with advanced NSCLC without driver mutations according to their condition. For second or more lines, ramucirumab plus docetaxel (RD) is a leading treatment option and is superior to docetaxel alone, as confirmed in a previous phase III study.^5^ Using real‐world data, we have shown that RD is a suitable second‐line treatment for patients with advanced NSCLC after combined chemotherapy/PD‐1 blockade therapy.^6^ Currently, there are no established parameters for predicting the clinical benefit of RD after first‐line chemoimmunotherapy.

Ramucirumab is an antivascular endothelial growth factor (VEGF) receptor 2 (VEGFR2) fully human monoclonal IgG1 antibody that inhibits tumor growth by blocking the interaction of VEGFR2 with its natural ligand.^7^ No study reporting the efficacy of RD in previously treated NSCLC has described VEGFR2 expression as a potential prognostic marker.

The current standard front‐line treatment for NSCLC patients without driver mutations includes PD‐1 blockade, with most patients receiving RD after PD‐1 blockade. Several recent studies have shown that RD is more effective when administered immediately after regimens with versus without PD‐1 blockade.^8^, ^9^, ^10^, ^11^ Moreover, an exploratory analysis in a phase III study of RD in patients with stage IV NSCLC suggests that RD may be effective after disease progression on platinum‐based regimens including taxane, pemetrexed, gemcitabine, or bevacizumab.^12^ In contrast, second‐line RD treatment was not beneficial in patients with advanced NSCLC and a *KRAS* mutation; whether prior chemotherapy which included an ICI affected RD efficacy was not addressed in that study.^13^ Several researchers have reported real‐world data on RD in patients with previously treated NSCLC in large‐scale retrospective studies^14^, ^15^; in these studies the front‐line platinum‐based chemotherapy included immunotherapy.

Overall, whether prior chemoimmunotherapy affects the efficacy of RD and the mechanism remains unclear. Prior immunotherapy may enhance responsiveness to RD via a mechanism involving synergism between the VEGFR2 signaling pathway and the tumor immune environment. Our REACTIVE study examined the relationship between front‐line chemoimmunotherapy and RD efficacy,^6^ and we expect that our approach will explain why RD is an appropriate choice after chemoimmunotherapy. Additionally, further investigations are needed to identify markers that accurately predict RD efficacy after chemoimmunotherapy.

To address these issues, we conducted a retrospective study to predict the clinical benefit of RD treatment after front‐line chemoimmunotherapy in patients with advanced NSCLC, using patient population from our REACTIVE study.^6^

## METHODS

### Patients and study design

The REACTIVE study design has been reported previously.^6^ The REACTIVE study was a multicenter retrospective investigation involving 62 Japanese institutions.

It included 288 patients (222 men and 66 women; median age, 67 years; age range, 20–82 years) with advanced NSCLC who received platinum‐based chemotherapy plus anti‐PD‐1/PD‐L1 antibodies as first‐line therapy and RD as second‐line treatment between January 2017 and August 2020. Clinical data were extracted from medical records, and the sample in the present study was the same as that of the REACTIVE study.^6^

This study was approved by the Institutional Ethics Committee of the International Medical Center at Saitama Medical University. The requirement for written informed consent was waived by the committee because of the retrospective nature of the study.

### Treatment and assessment

All patients received platinum‐based chemotherapy with anti‐PD‐1/PD‐L1 antibodies. The IMpower 150 (1200 mg atezolizumab, 15 mg/kg bevacizumab, area under the concentration‐time curve of 5 mg/mL per min carboplatin, and 1750 mg/m^2^ paclitaxel), KEYNOTE 189 (area under the concentration‐time curve of 5 mg/mL per min carboplatin, 500 mg/m^2^ pemetrexed, and 200 mg pembrolizumab), and KEYNOTE 407 (area under the concentration‐time curve of 5 mg/mL per min carboplatin, 100 mg/m^2^ nab‐paclitaxel, and 200 mg pembrolizumab) regimens were intravenously administered.^2^, ^3^, ^4^ The results of physical examinations, complete blood counts, and biochemical tests were assessed by the chief physician. Toxicity was graded based on the Common Terminology Criteria for Adverse Events version 4.0. Tumor response was evaluated according to the Response Evaluation Criteria in Solid Tumors version 1.1.^16^

### Statistical analysis

The statistical significance level was set at *p* < 0.05. Fisher's exact test was used to examine the association between two categorical variables. Progression‐free survival (PFS) was defined as the time from RD initiation to disease progression or death. Overall survival (OS) was defined as the time from RD initiation to death from any cause. The Kaplan–Meier method was used to estimate survival as a function of time, and survival differences were analyzed using the log‐rank test. Univariate and multivariate analyses of different variables were performed using logistic regression.

In our post hoc analysis, the objective response rate (ORR), disease control rate (DCR), PFS, and OS for RD were evaluated according to the presence or absence of maintenance therapy, duration of ICI treatment, and ORR of front‐line platinum‐based chemotherapy plus anti‐PD‐1/PD‐L1 antibodies. For further analysis, the cutoff value for the duration of ICI treatment was defined as median values. In our exploratory analysis, moreover, receiver operating characteristic (ROC) curve analysis was performed to confirm the optimal cutoff value of the duration of ICI treatment, and the sensitivity and specificity were calculated to determine the optimal cutoff value for differentiating responders from nonresponders by the ROC curve. Responders were defined as those with a PFS of >6 months for DR. Those variables with *p* < 0.05 in univariable analyses were fit in a multivariable model. The duration of ICI treatment was defined as the period from the starting point of induction therapy by chemoimmunotherapy to the discontinuation of ICI therapy. All statistical analyses were performed using GraphPad Prism (version 8.0; GraphPad Software) and JMP 14.0 (SAS Institute Inc.).

## RESULTS

### Patient demographics and front‐line therapy

Patient information including maintenance therapy, duration of ICI treatment, and ORR of front‐line chemoimmunotherapy are shown in Table 1. As described in our previous report,^6^ KEYNOTE 189,^2^ KEYNOTE 407,^3^ IMpower 150,^4^ IMpower 130,^17^ IMpower 132,^18^ and other regimens were administered to 160 (55.6%), 58 (20.1%), 15 (5.2%), four (1.4%), 11 (3.8%), and 38 (13.2%) patients, respectively. The median number of cycles of induction chemotherapy was four (range, 1–8 cycles). The median number of cycles of maintenance therapy after induction chemotherapy was four (range, 1–29 cycles). The median duration of ICI treatment was 180 days (range, 0–671 days). Therefore, the cutoff value for the duration of ICI treatment were 180 days.

**TABLE 1:** Patient characteristics according to the efficacy of front‐line therapy.

Variables		Total *N* (%)	Maintenance therapy			Duration of ICI treatment			Objective response rate		
			Yes *N* = 225	No *N* = 63	*p*‐value	>180 days *N* = 108	≤180 days *N* = 180	*p*‐value	CR or PR^a^ *N* = 154	SD or PD^a^ *N* = 127	*p*‐value
Age, years	<75	262 (91%)	205	57	0.808	96	166	0.396	139	116	0.837
	≥75	26 (9%)	20	6		12	14		15	11	
Gender	Male	222 (77%)	174	48	0.866	85	137	0.665	121	95	0.479
	Female	66 (23%)	51	15		23	43		33	32	
PS	0–1	269 (93%)	211	58	0.576	104	165	0.147	145	117	0.634
	2–3	19 (7%)	14	5		4	15		9	10	
Histology	Ad	199 (69%)	156	43	0.878	74	125	0.895	95	98	**0.006**
	Non‐Ad	89 (31%)	69	20		34	55		59	29	
Smoking history	Yes	237 (82%)	182	55	0.268	88	149	0.873	129	101	0.437
	No	51 (18%)	43	8		20	31		25	26	
Clinical staging	III/IV	236 (82%)	182	54	0.460	87	149	0.638	132	99	0.116
	Ope rec.	52 (18%)	43	9		21	31		22	28	
PD‐L1^a^ (%)	≥50	60 (23%)	46	14	0.192	22	38	**<0.001**	37	23	0.365
	1–49	81 (31%)	68	13		57	24		43	36	
	<1	123 (46%)	90	33		39	84		60	59	
ILD	Yes	6 (2%)	6	0	0.345	3	3	0.676	3	3	>0.999
	No	282 (98%)	219	63		105	177		151	124	
Radiation therapy	Yes	92 (32%)	62	30	**0.003**	24	68	**0.006**	43	48	0.095
	No	196 (68%)	163	33		84	112		111	79	
Pleural effusion	Yes	42 (15%)	38	4	**0.042**	19	23	0.301	24	15	0.390
	No	246 (85%)	187	59		89	157		130	112	
Brain metastasis	Yes	40 (14%)	28	12	0.215	13	27	0.598	16	22	0.114
	No	248 (86%)	197	51		95	153		138	105	
Liver metastasis	Yes	34 (12%)	23	11	0.124	9	23	0.332	18	15	>0.999
	No	254 (88%)	202	52		99	157		136	112	
Bone metastasis	Yes	97 (34%)	69	28	**0.049**	33	64	0.440	53	42	0.899
	No	191 (66%)	156	35		75	116		101	85	

*Note*: Bold indicates statistically significance.

Abbreviations: Ad, adenocarcinoma; CR, complete response; ICI, immune checkpoint inhibitor; ILD, having history of interstitial lung disease associated at initial diagnosis; N, number of patients; Ope rec., postoperative recurrence; PD, progressive disease; PD‐L1, programmed death ligand‐1; PR, partial response; PS, performance status; SD, stable disease.

^a^ Not evaluable cases are excluded from analysis; radiation therapy, patients receiving any radiation therapy before ramucirumab plus docetaxel.

The anti‐PD‐1 and anti‐PD‐L1 antibodies used for chemoimmunotherapy were detected in 236 (81.9%) and 52 (18.1%) patients, respectively. Among the 288 patients, 225 (78.1%) received maintenance therapy after induction chemotherapy, and 108 (37.5%) received both maintenance therapy and ICIs for >180 days. On the other hand, all (100%) of 108 patients having ICIs for >180 days received maintenance therapy. Prior radiation therapy was frequently observed in patients receiving maintenance therapy or >180 days of ICI treatment. Pleural effusion and bone metastases were associated with the presence of maintenance therapy. Negative PD‐L1 expression was closely associated with an ICI treatment duration of ≤180 days. Nonadenocarcinoma (AC) significantly correlated with a complete response (CR) or partial response (PR) to front‐line treatment.

### Efficacy of front‐line therapy

Among the 288 patients in our study, CR, PR, stable disease (SD), progressive disease (PD), and not evaluable (NE) were observed in one, 153, 87, 40, and seven patients, respectively. The ORR and DCR of front‐line treatment were 54.8% and 85.7%, respectively. Among the patients receiving RD, CR, PR, SD, PD, and NE were observed in one, 82, 118, 73, and 14 patients, respectively. The ORR and DCR of RD were 28.8% and 69.8%, respectively.

Table 2 shows the ORR and DCR of RD according to the presence/absence of maintenance therapy, duration of ICI treatment, and ORR of front‐line chemoimmunotherapy. Maintenance therapy significantly improved the DCR of RD regardless of the histological type, but not the ORR. Continuous administration of an ICI for >180 days significantly improved the DCR. The CR or PR of front‐line therapy significantly increased the ORR of RD, regardless of histology.

**TABLE 2:** Efficacy of ramucirumab plus docetaxel according to the efficacy of front‐line therapy.

Variables		Efficacy	Front‐line chemoimmunotherapy								
			Maintenance therapy			Duration of ICI treatment			Objective response rate		
			Yes	No	*p*‐value	>180 days	≤180 days	*p*‐value	CR or PR	SD or PD	*p*‐value
Ramucirumab plus docetaxel	All patients										
	*N* = 274		*N* = 214	*N* = 60		*N* = 103	*N* = 171		*N* = 147	*N* = 125	
	ORR	28.8%	32.7% (70/214)	21.7% (13/60)	0.113	33.9% (35/103)	27.4% (47/171)	0.277	39.4% (58/147)	19.2% (24/125)	**<0.001**
	DCR	69.8%	78.5% (168/214)	55.0% (33/60)	**<0.001**	84.4% (87/103)	66.6% (114/171)	**0.001**	78.9% (116/147)	66.4% (83/125)	**0.027**
	Adenocarcinoma										
	*N* = 187		*N* = 147	*N* = 40		*N* = 70	*N* = 117		*N* = 89	*N* = 96	
	ORR	33.8%	35.3% (52/147)	27.5% (11/40)	0.450	38.5% (27/70)	30.7% (36/117)	0.337	44.9% (40/89)	22.9% (22/96)	**0.001**
	DCR	77.4%	80.9% (119/147)	62.5% (25/40)	**0.019**	84.2% (59/70)	72.6% (85/117)	0.074	83.1% (74/89)	70.8% (68/96)	0.563
	Nonadenocarcinoma										
	*N* = 87		*N* = 67	*N* = 20		*N* = 33	*N* = 54		*N* = 58	*N* = 29	
	ORR	22.9%	26.8% (18/67)	10.0% (2/20)	0.140	27.2% (9/33)	20.3% (11/54)	0.600	31.0% (18/58)	6.8% (2/29)	**0.014**
	DCR	65.5%	73.1% (49/67)	40.0% (8/20)	**0.014**	84.8% (28/33)	53.7% (29/54)	**0.004**	72.4% (42/58)	51.7% (15/29)	0.092

*Note*: Not evaluable (NE) cases in front‐line and ramucirumab plus docetaxel were excluded from analysis. Bold indicates statistically significance.

Abbreviations: CR, complete response; DCR, disease control rate; ICI, immune checkpoint inhibitor; N, number of patients; ORR, objective response rate; PD, progressive disease; PR, partial response; SD, stable disease.

### Univariate and multivariate survival analysis for predictors of RD efficacy

**FIGURE 1:**
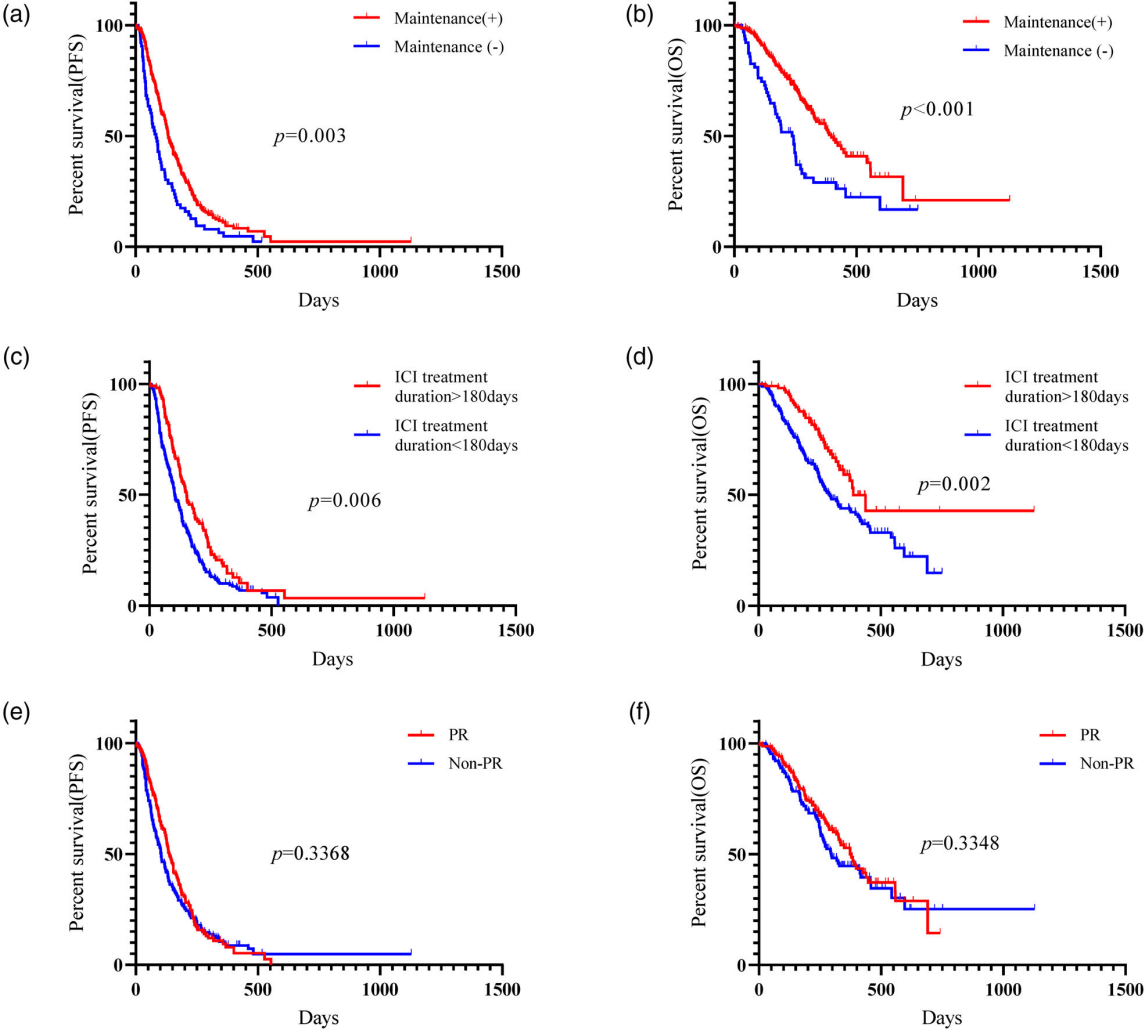
Kaplan–Meier survival curve of ramucirumab plus docetaxel (RD) according to the presence or absence of maintenance therapy, immune checkpoint inhibitor (ICI) treatment duration by cutoff of 180 days, and objective response of front‐line chemoimmunotherapy in all patients. Progression‐free survival (PFS) (a) and overall survival (OS) (b) for RD were significantly longer in patients with maintenance therapy than in those without maintenance therapy. The patients who received RD with ICI treatment duration of more than 180 days achieved a significantly better PFS (c) and OS (d) than those with less than 180 days. No statistically significant difference in the PFS (e) and OS (f) for RD was observed in between the patients with partial response (PR) and non‐PR.

The median PFS and OS times for all patients were 4.1 and 11.6 months, respectively. A total of 260 patients experienced disease progression, 146 of whom died. Univariate analysis of all patients identified performance status, histology, maintenance therapy, and ICI treatment duration >180 days as significant predictors of better PFS and OS after RD administration (log‐rank test, Table 3). Multivariate analysis confirmed that these factors independently predicted favorable PFS and OS (Table 3). The Kaplan–Meier survival curves for PFS and OS according to maintenance therapy, ICI treatment duration, and objective response are shown in Figure 1. Although the survival analysis according to histology was displayed in Figure A1 (online only), the maintenance and ICI treatment duration >180 days were closely associated with favorable prognosis regardless of histological type. The DCR of front‐line chemoimmunotherapy was also related to the outcome after RD administration in all patients (Figure A2, online only).

**TABLE 3:** Univariate and multivariate survival analysis.

Variables		Progression‐free survival					Overall survival				
		Univariate analysis		Multivariate analysis			Univariate analysis		Multivariate analysis		
		MST (d)	*p*‐value	HR	95% CI	*p*‐value	MST (d)	*p*‐value	HR	95% CI	*p*‐value
Age (years)	<75	124	0.537				373	0.734			
	≥75	127					317				
Gender	Male	124	0.577				332	0.455			
	Female	123					388				
PS	0–1	126	**0.011**	1.392	1.108–1.737	**0.011**	373	**<0.001**	3.472	1.804–6.083	**<0.001**
	2–3	41					127				
Histology	Ad	134	**0.005**	1.250	1.095–1.422	**0.001**	416	**0.003**	1.729	1.224–2.421	**0.002**
	Non‐Ad	91					267				
Smoking history	Yes	123	0.822				326	0.367			
	No	133					397				
Clinical staging	III/IV	124	0.328				326	0.072			
	Ope rec.	141					690				
Maintenance therapy^a^	Yes	216	**0.003**	1.182	1.010–1.378	**0.037**	397	**<0.001**	1.754	1.185–2.578	**0.005**
	No	63					236				
Duration of ICI treatment^a^	>180 days	152	**0.006**	1.153	1.007–1.321	**0.038**	388	**0.002**	1.494	1.003–2.250	**0.048**
	≤180 days	103					288				
Objective response^a^	CR or PR	135	0.695				378	0.419			
	SD or PD	103					298				

*Note*: Bold indicates statistically significance.

Abbreviations: 95% CI, 95% confidence interval; Ad, adenocarcinoma; CR, complete response; d, days; ICI, immune checkpoint inhibitor; MST, median survival time (days); Ope rec., postoperative recurrence; PD, progressive disease; PD‐L1, programmed death ligand‐1; PR, partial response; PS, performance status; SD, stable disease.

^a^ Therapeutic status of front‐line treatment.

The effects of RD on PFS and OS were unaffected by PD‐L1 expression levels (Figure A3, online only). As shown in Figure A4 (online only), the number of cycles of maintenance therapy (>4 vs. 1–4) significantly altered the effects of RD on PFS in all and non‐AC patients; however, it did not alter the effects of RD on OS regardless of histology.

## DISCUSSION

Our exploratory analysis identified the transition to maintenance therapy and ICI treatment for >180 days in front‐line chemoimmunotherapy as independent predictors of better outcomes after RD administration in patients with advanced NSCLC. Although all patients receiving ICIs for >180 days also received maintenance therapy, sensitivity to prior immunotherapy appeared to be associated with increased RD efficacy. In agreement, in previous studies, prior ICI treatment was a critical determinant of an improved response to RD.^8^, ^9^, ^10^, ^11^ At least, continuous administration of ICI for >180 days as prior treatment could definitely predict the PFS of >6 months for DR.

The results of our large‐scale study suggest that RD is an appropriate second‐line choice for patients with advanced NSCLC whose front‐line treatment included maintenance therapy and continuous administration of an ICI. The present study does not explain how maintenance therapy including continuous ICI treatment improved treatment outcome after RD administration.

In general, ICIs promote the entry of CD4, CD8, and other tumor‐infiltrating lymphocytes (TILs) into tumors, where they subsequently kill tumor cells.^19^ Angiogenetic proteins such as vascular endothelial growth factor (VEGF) and VEGFR create an immunosuppressive tumor microenvironment, with resultant increases in the expression of FOXP3 and the number of myeloid‐derived suppressor cells.^20^, ^21^, ^22^ We hypothesize that extended ICI treatment achieves a tumor microenvironment in which CD4/CD8 TIL levels are high and FOXP3 levels are low, contributing to the downregulation of VEGF signaling. In this situation, tumor growth may be easily suppressed by exposure to VEGFR2 inhibitors such as ramucirumab. In contrast, patients with low sensitivity to ICIs may be resistant to VEGFR2 inhibitors owing to upregulation of VEGF signaling in response to increased FOXP3 expression.

Recently, we reported that high VEGFR2 expression in tumor tissue significantly predicted a poor outcome after PD‐1 blockade and closely correlated with the number of FOXP3‐expressing TILs in patients with advanced NSCLC.^22^ In the present study, we did not investigate whether the CD4/CD8 TIL and FOXP3 levels in the tumor specimens correlated with VEGFR2 expression, and the biological mechanism underlying the increased efficacy of RD after continuous ICI treatment for >180 days remains unresolved. Further studies are needed to elucidate the relationship between the efficacy of VEGFR2 inhibitors and the status of the tumor microenvironment after ICI administration.

In our previous study on 288 patients with advanced NSCLC, RD after first‐line chemoimmunotherapy had an ORR of 28.8%, DCR of 69.8%, median PFS time of 4.1 months, and median OS time of 11.6 months.^6^ In the study by Brueckl et al. on 77 NSCLC patients, these values were 32.5%, 62.4%, 6.4 months, and 15.5 months, respectively.^13^ In the study by Ishida et al. on 18 NSCLC patients, the ORR of RD was 55.6% and the PFS time was 5.8 months.^23^ They found that the patients who responded to prior chemoimmunotherapy for more than 8.8 months exhibited a significantly longer response to RD than those responded for less than 8.8 months.^23^ This is similar to the results of our study; however, the duration of response was different (8.8 vs. 6.0 months). However, their study was small sample size with 18 patients, compared to our large sample with 288 patients. At least, the duration responding to prior chemoimmunotherapy may be important for the efficacy of RD as second line setting. In the current analysis, the patients who received maintenance therapy including ICI treatment achieved an ORR of 32.7%, DCR of 78.5%, PFS time of 7.7 months (216 days), and OS time of 14.2 months (397 days). Although it is difficult to compare the results of individual studies because of different sample sizes, we suggest that sensitivity to chemoimmunotherapy may engender a clinical benefit of RD in the second‐line setting.

To date, there is no evidence to as to whether prior administration of cytotoxic agents improves the efficacy of RD in various human neoplasms. Thus, we believe that maintenance therapy should include continuous ICI treatment for a sufficient amount of time. Tumor shrinkage due to chemoimmunotherapy has been shown to significantly increase the ORR of RD but not to improve the treatment outcome. It also makes it difficult to distinguish the contribution of cytotoxic agents from that of ICIs. In our study, the response rate of chemoimmunotherapy did not predict the outcome of subsequent RD treatment.

Our study had several limitations. First, VEGFR2 expression in tumor cells was not assessed. Thus, whether VEGFR2 expression predicts RD efficacy remains unclear, and further investigation is required. Second, different chief physicians chose the first‐line chemotherapy regimens, and differences in the regimens (albeit small) might have biased our results. Finally, because all patients received RD as the second‐line treatment after chemoimmunotherapy, we could not compare the outcomes of RD with those of ramucirumab or docetaxel alone. Thus, it remains unclear whether prior ICI treatment affects the efficacy of ramucirumab, docetaxel, or both. Prospective studies are needed to address this issue. A previous study reported an improved response rate to docetaxel alone after ICI treatment^24^; however, the underlying mechanism remains unclear.

In conclusion, extended ICI treatment after chemoimmunotherapy and maintenance therapy enhanced the efficacy of second‐line RD treatment in patients with advanced NSCLC. Sensitivity to ICI therapy may improve the outcomes of RD treatment.

## AUTHOR CONTRIBUTIONS

Ou Yamaguchi: Conceptualization; Data curation; Formal analysis; Funding acquisition; Investigation; Methodology; Project administration; Writing original draft; Draft review and editing. Keita Mori: Conceptualization; Formal analysis; Methodology; Writing original draft; Writing original draft; Draft review and editing. Saori Takata: Investigation; Draft review and editing. Kazuhiko: Investigation; Draft review and editing. Kenichi Chikamori: Investigation; Draft review and editing. Nozomu Kimura: Investigation; Draft review and editing. Yoshiaki Nagai: Investigation; Draft review and editing. Taku Nakagawa: Investigation; Draft review and editing. Satoshi Igawa: Investigation; Draft review and editing. Taishi Harada: Investigation; Draft review and editing. Hiroshige Yoshioka: Investigation; Draft review and editing. Hisashi Tanaka: Investigation; Draft review and editing. Hitomi Nogawa: Investigation; Draft review and editing. Hiroaki Satoh: Investigation; Draft review and editing. Toshihiro Shiozawa: Investigation; Draft review and editing. Kosuke Tsuji: Investigation; Draft review and editing. Kunihiko Kobayashi: Conceptualization; Methodology; Draft review and editing. Kyoichi Kaira: Conceptualization; Funding acquisition; Methodology; Project administration; Writing original draft; Draft review and editing.

## FUNDING INFORMATION

This study was financially supported by Eli Lilly Japan K.K.

## CONFLICT OF INTEREST STATEMENT

Kyoichi Kaira reports a relationship with AstraZeneca Pharmaceuticals LP that includes speaking and lecture fees. Kunihiko Kobayashi reports a relationship with AstraZeneca that includes speaking and lecture fees. Kunihiko Kobayashi reports a relationship with Takeda Pharmaceutical Co Ltd that includes speaking and lecture fees. peaking and lecture fees. Ou Yamaguchi reports a relationship with Eli Lilly Japan K.K. that includes speaking and lecture fees. Ou Yamaguchi reports a relationship with Ono Pharmaceutical Co Ltd that includes speaking and lecture fees. Ou Yamaguchi reports a relationship with Bristol Myers Squibb Co that includes speaking and lecture fees. Ou Yamaguchi reports a relationship with Chugai Pharmaceutical Co Ltd that includes speaking and lecture fees. Ou Yamaguchi reports a relationship with Merck Sharp & Dohme Corp that includes speaking and lecture fees. Ou Yamaguchi reports a relationship with AstraZeneca that includes speaking and lecture fees.

## DISCLAIMER

The content of this publication does not necessarily reflect the views of policies of the Department of Health and Human Services, nor does mention of trade names, c.

## Supporting information


**Figure A1.** Kaplan–Meier survival curve of ramucirumab plus docetaxel (RD) according to the presence or absence of maintenance therapy and ICI treatment duration by cutoff of 180 days of front‐line chemo‐immunotherapy in patients with adenocarcinoma (AC) and nonadenocarcinoma (non‐AC). The AC patients receiving maintenance therapy tended to be longer progression free survival (PFS) rather than those without maintenance therapy (a). PFS for RD was significantly longer non‐AC (c) patients with maintenance therapy than in those without maintenance therapy. Overall survival (OS) for RD yielded a significantly longer in AC (b), and non‐AC (d) patients than in those without maintenance therapy. The AC patients who received RD with ICI treatment duration of more than 180 days achieved a significantly better PFS (e) and OS (f) than those with less than 180 days. PFS for RD was significantly longer in patients with non‐AC receiving ICI treatment of more than 180 days than in those with less than 180 days (g), but not OS (h).

**Figure A2.** Kaplan–Meier survival curve according to objective response. By the survival analysis according to CR/PR, stable disease (SD) and progressive disease (PD), progression‐free survival (PFS) (a) and overall survival (OS) (b) were significantly different between all patients with CR/PR/SD and PD. The same survival analysis was performed in adenocarcinoma (AC) (c; PFS and d; OS) and non‐adenocarcinoma (non‐AC) (g; PFS and h; OS) patients.

**Figure A3.** Kaplan–Meier survival curve according to PD‐L1 expression (>50%, 1%–49%, and 0%). No statistically significant difference in the progression‐free survival (PFS) (a) and overall survival (OS) (b) for ramucirumab plus docetaxel (RD) was observed.

**Figure A4.** Kaplan–Meier survival curve according to maintenance therapy with >4 and 1–4 cycles. The progression‐free survival (PFS) and overall survival (OS) in all (a, b), adenocarcinoma (AC) (c, d), and nonadenocarcinoma (non‐AC) (e, f) patients were presented.

## Data Availability

Data are available on reasonable request.
