# Supplementary figures and images for: Extended ICI treatment after first‐line chemoimmunotherapy could predict the clinical benefit of ramucirumab plus docetaxel in advanced non‐small lung cancer: Post hoc analysis from NEJ051 (REACTIVE study)
Source: Thorac Cancer. 2023 Nov 27;15(2):163–71. doi: 10.1111/1759-7714.15173 (PMC10788474; doi:10.1111/1759-7714.15173)

## Slide 1
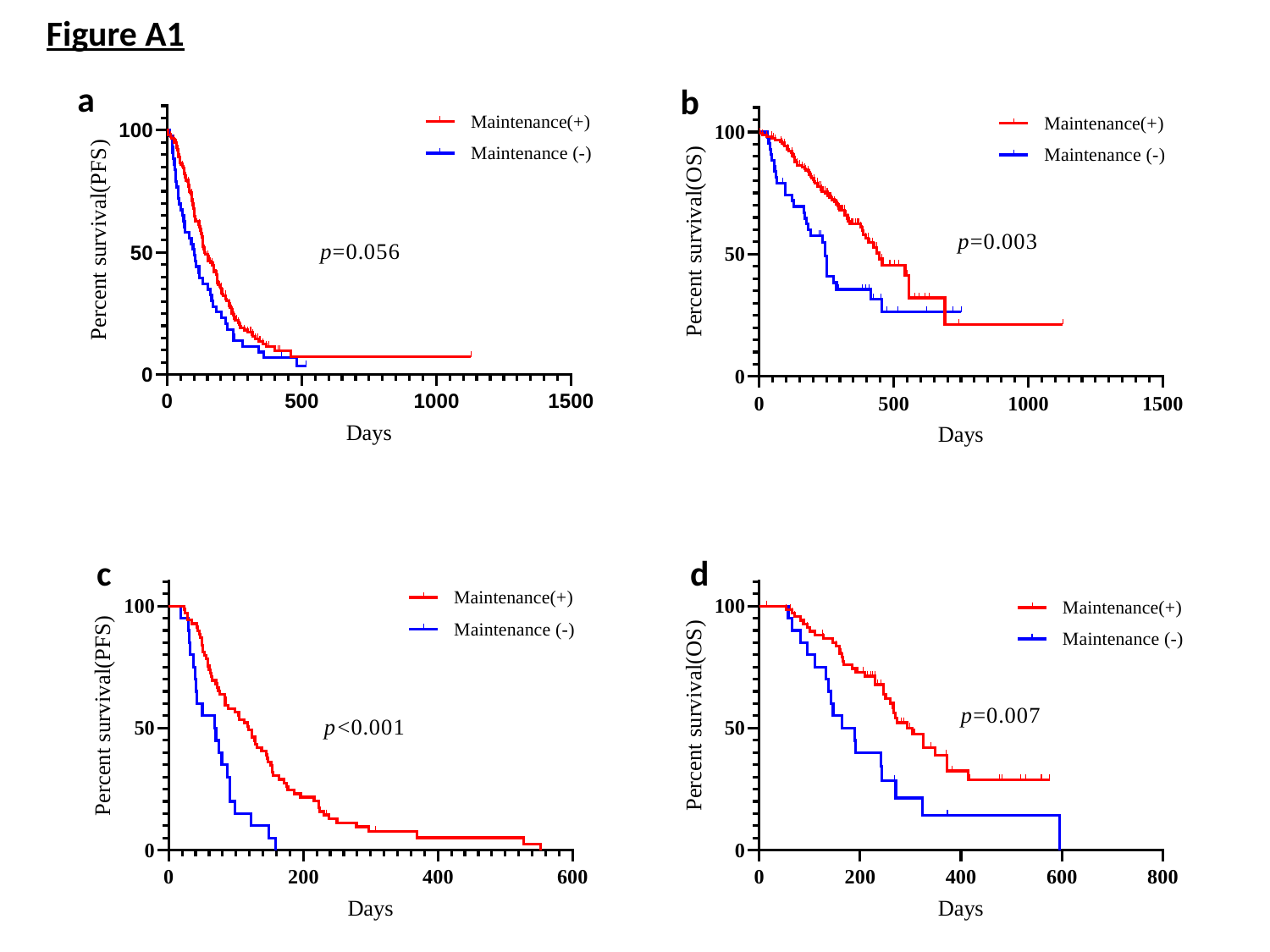

Figure A1
a
b
c
d

## Slide 2
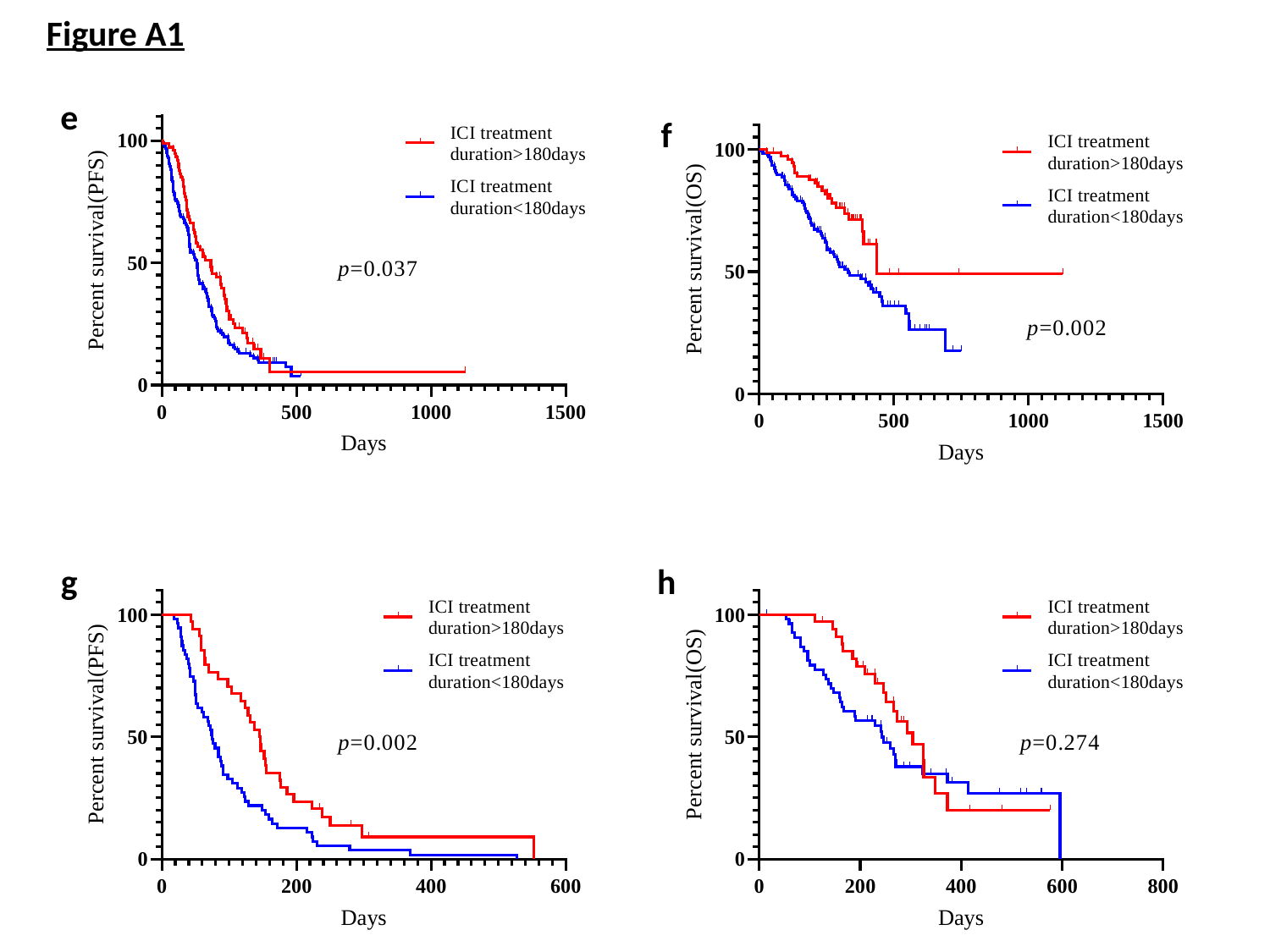

Figure A1
e
f
g
h

Supplement: Supplementary file 1 — Figure A1. Kaplan–Meier survival curve of ramucirumab plus docetaxel (RD) according to the presence or absence of maintenance therapy and ICI treatment duration by cutoff of 180 days of front‐line chemo‐immunotherapy in patients with adenocarcinoma (AC) and nonadenocarcinoma (non‐AC). The AC patients receiving maintenance therapy tended to be longer progression free survival (PFS) rather than those without maintenance therapy (a). PFS for RD was significantly longer non‐AC (c) patients with maintenance therapy than in those without maintenance therapy. Overall survival (OS) for RD yielded a significantly longer in AC (b), and non‐AC (d) patients than in those without maintenance therapy. The AC patients who received RD with ICI treatment duration of more than 180 days achieved a significantly better PFS (e) and OS (f) than those with less than 180 days. PFS for RD was significantly longer in patients with non‐AC receiving ICI treatment of more than 180 days than in those with less than 180 days (g), but not OS (h). [file TCA-15-163-s003.pptx]

## Slide 1
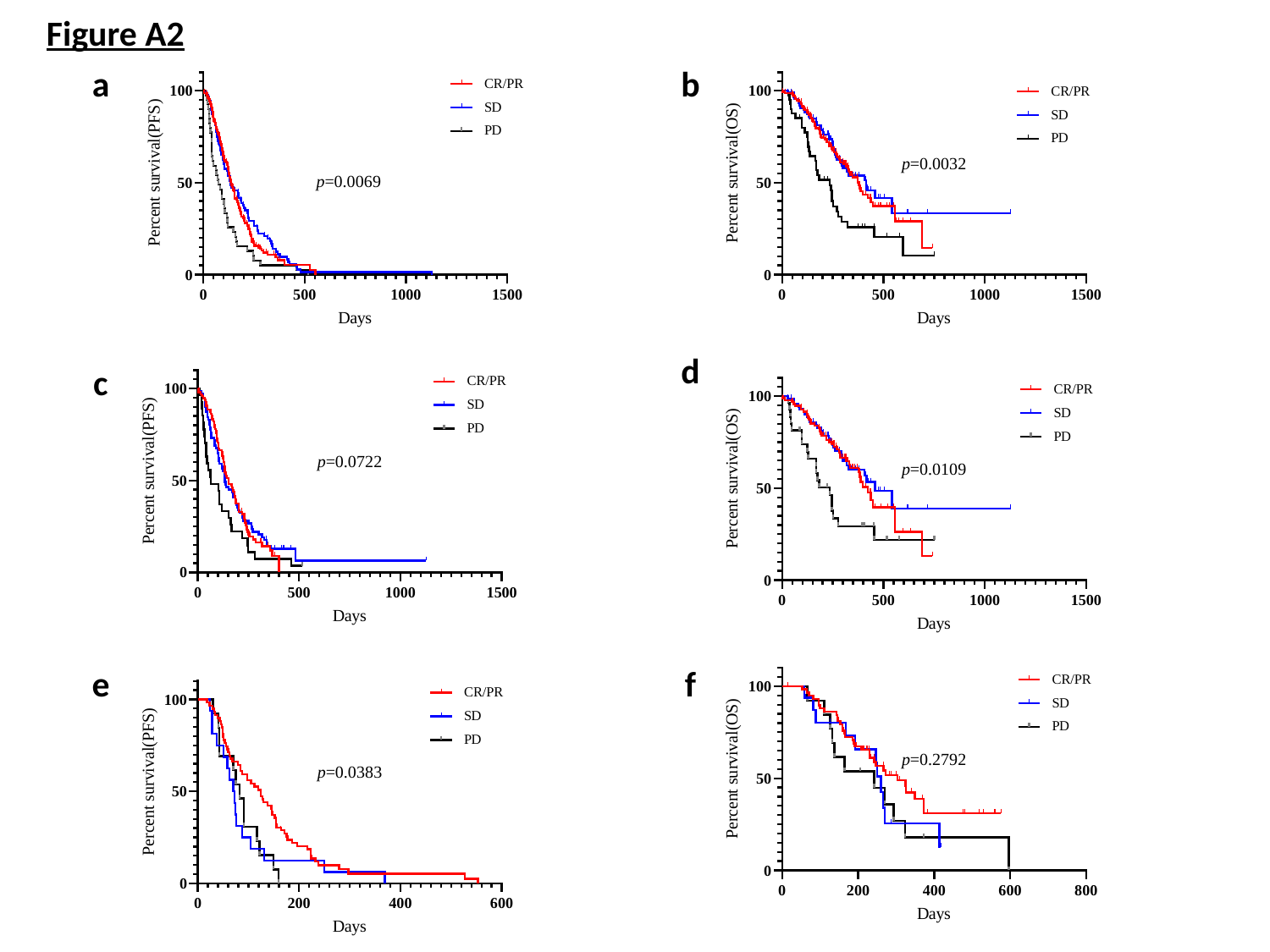

Figure A2
a
b
d
c
e
f

Supplement: Supplementary file 2 — Figure A2. Kaplan–Meier survival curve according to objective response. By the survival analysis according to CR/PR, stable disease (SD) and progressive disease (PD), progression‐free survival (PFS) (a) and overall survival (OS) (b) were significantly different between all patients with CR/PR/SD and PD. The same survival analysis was performed in adenocarcinoma (AC) (c; PFS and d; OS) and non‐adenocarcinoma (non‐AC) (g; PFS and h; OS) patients. [file TCA-15-163-s001.pptx]

## Slide 1
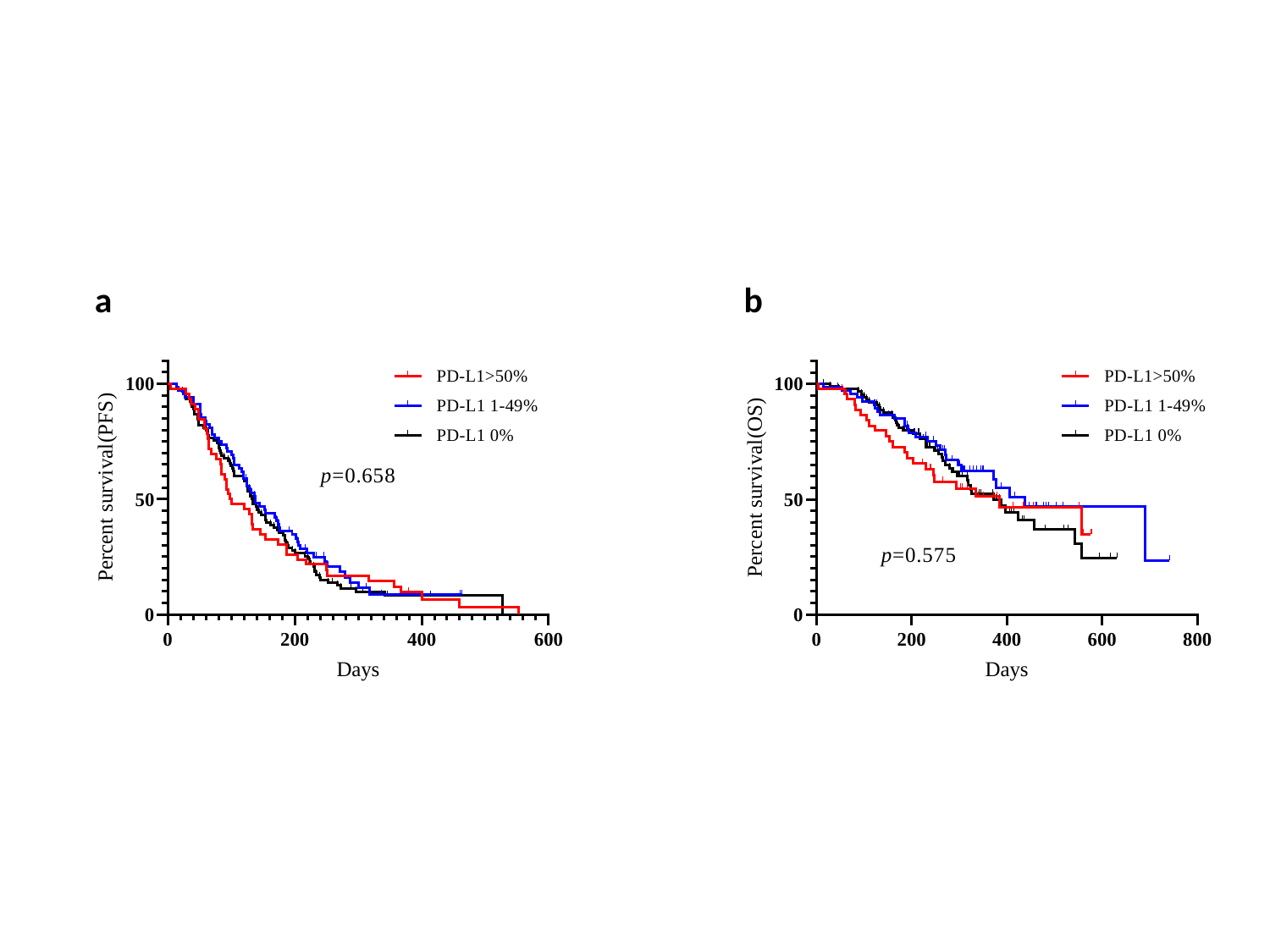

a
b

Supplement: Supplementary file 3 — Figure A3. Kaplan–Meier survival curve according to PD‐L1 expression (>50%, 1%–49%, and 0%). No statistically significant difference in the progression‐free survival (PFS) (a) and overall survival (OS) (b) for ramucirumab plus docetaxel (RD) was observed. [file TCA-15-163-s002.pptx]

## Slide 1
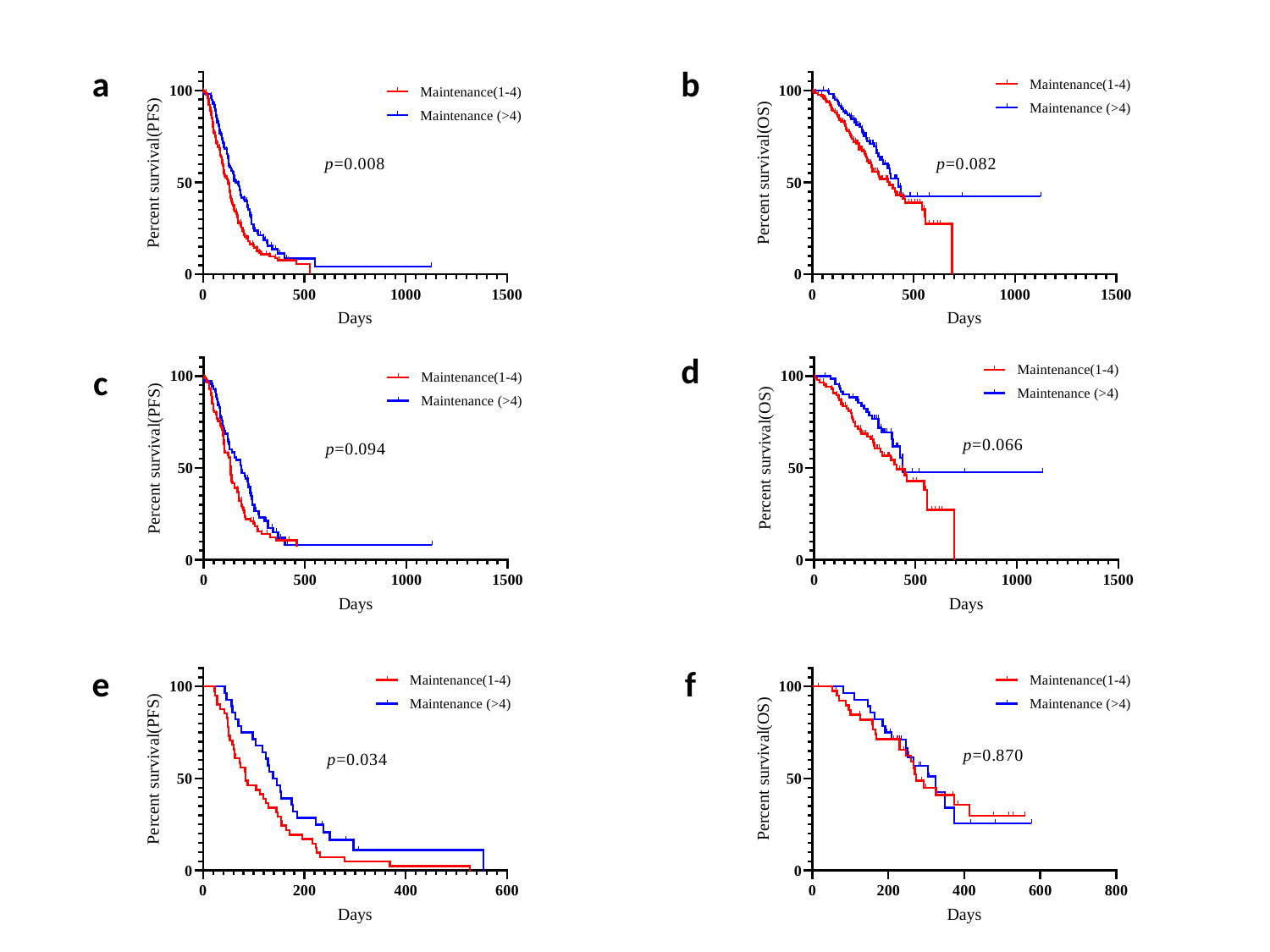

a
b
d
c
e
f

Supplement: Supplementary file 4 — Figure A4. Kaplan–Meier survival curve according to maintenance therapy with >4 and 1–4 cycles. The progression‐free survival (PFS) and overall survival (OS) in all (a, b), adenocarcinoma (AC) (c, d), and nonadenocarcinoma (non‐AC) (e, f) patients were presented. [file TCA-15-163-s004.pptx]
